# Epithelial‐mesenchymal transition in cancer metastasis through the lymphatic system

**DOI:** 10.1002/1878-0261.12092

**Published:** 2017-06-26

**Authors:** Mikael C. Karlsson, Santiago F. Gonzalez, Josefin Welin, Jonas Fuxe

**Affiliations:** ^1^ Department of Microbiology, Tumor Biology and Cell Biology (MTC) Karolinska Institutet Stockholm Sweden; ^2^ Institute for Research in Biomedicine (IRB) Bellinzona Switzerland

**Keywords:** chemokines, EMT, immune cell properties, lymph metastasis, targeted migration, TGF‐β

## Abstract

It was already in the 18th century when the French surgeon LeDran first noted that breast cancer patients with spread of tumor cells to their axillary lymph nodes had a drastically worse prognosis than patients without spread (LeDran *et al*., 1752). Since then, metastatic spread of cancer cells to regional lymph nodes has been established as the most important prognostic factor in many types of cancer (Carter *et al*., 1989; Elston and Ellis, 1991). However, despite its clinical importance, lymph metastasis remains an underexplored area of tumor biology. Fundamental questions, such as when, how, and perhaps most importantly, why tumor cells disseminate through the lymphatic system, remain largely unanswered. Accordingly, no treatment strategies exist that specifically target lymph metastasis. The identification of epithelial‐mesenchymal transition (EMT) as a mechanism, which allows cancer cells to dedifferentiate and acquire enhanced migratory and invasive properties, has been a game changer in cancer research. Conceptually, EMT provides an explanation for why epithelial cancers with poor differentiation status are generally more aggressive and prone to metastasize than more differentiated cancers. Inflammatory cytokines, such as TGF‐β, which are produced and secreted by tumor‐infiltrating immune cells, are potent inducers of EMT. Thus, reactivation of EMT also links cancer‐related inflammation to invasive and metastatic disease. Recently, we found that breast cancer cells undergoing TGF‐β‐induced EMT acquire properties of immune cells allowing them to disseminate in a targeted fashion through the lymphatic system similar to activated dendritic cells during inflammation. Here, we review our current understanding of the mechanisms by which cancer cells spread through the lymphatic system and the links to inflammation and the immune system. We also emphasize how imaging techniques have the potential to further expand our knowledge of the mechanisms of lymph metastasis, and how lymph nodes serve as an interface between cancer and the immune system.

**Abbreviations**
APCs: antigen‐presenting immune cells
CTL: cytotoxic T lymphocytes
CXADR: coxsackie and adenovirus receptor
DC: dendritic cells
EGFR: epidermal growth factor receptor
EMT: epithelial‐mesenchymal transition
EndoMT: endothelial/mesenchymal transition
FDCs: follicular dendritic cells
IVM: intravital two‐photon microscopy
JAM‐A: junction adhesion molecule A
LN: lymph node
MDSC: myeloid‐derived suppressor cells
PECAM‐1: platelet endothelial cell adhesion molecule 1
SSM: subcapsular sinus macrophages
TAMs: tumor‐associated macrophages
TGF‐β: transforming growth factor beta
VEGF: vascular endothelial growth factor

## 1 Introduction

Metastasis is a complex process, during which cancer cells—for not completely known reasons, migrate away from the primary tumor, gain access to the circulation, and subsequently home to distant organs—most frequently the lungs and the liver, where they establish growth. Recent data indicate that the metastatic process is not entirely linked to tumor growth, *per se*, but is controlled by other factors and can occur from early lesions (Husemann *et al*., 2008). Although described as a rather inefficient process, the formation of distant metastases is what accounts for the largest numbers of deaths from cancer (Fidler and Balch, 1987; Luzzi *et al*., 1998). In contrast to locally growing tumors, which generally have better prognosis as they can be removed by surgical resection and/or by irradiation, metastatic cancers are significantly less treatable. Metastatic tumors tend to grow at multiple foci in distant organs and are difficult to remove surgically, or by irradiation. In addition, metastatic cancer cells are often resistant to treatment with chemotherapeutic drugs. This may be explained by the fact that most chemotherapeutic drugs have been developed to specifically target the proliferative capacity of cancer cells rather than their capacity to disseminate and grow at distant sites, which are the characteristic properties of metastatic cancer cells.

Metastatic spread of cancer cells requires dissemination via the systemic circulation. Circulating tumor cells can be found in patients with almost any types of cancer (Allard *et al*., 2004). However, the subject of how they get access to the circulation is still a matter of debate. Some tumor cells may intravasate into blood vessels within the tumor microenvironment and thereby get direct access to the circulation. Tumor blood vessels induced by overexpression of vascular endothelial growth factor (VEGF) often have discontinuous cell–cell junctions and incomplete coverage of mural cells (Baluk *et al*., 2005). As such, abnormal blood vessels in tumors have incomplete vascular walls and are leaky and may be used by tumor cells for intravasation. Yet, although angiogenic inhibitors targeting the VEGF pathway are quite efficient in blocking angiogenesis in mouse tumor models, they have shown limited effects on cancer progression and survival in human patients (Vasudev and Reynolds, 2014). In fact, it was shown in 2009 that although angiogenic inhibitors targeting the VEGF pathway block tumor growth in mouse models of pancreatic neuroendocrine carcinoma and glioblastoma, they concomitantly elicit tumor adaptation and progression toward higher malignancy grade (Paez‐Ribes *et al*., 2009), which was evident by increased lymphatic and distant metastasis.

Spread of cancer cells to regional lymph nodes is the most important prognostic factor in many types of cancer including breast cancer, head and neck cancer, prostate cancer, and colorectal cancer. In line with this, lymphatic invasion may be the predominant method of vascular invasion in breast cancer (Gujam *et al*., 2014; Mohammed *et al*., 2007). In head and neck cancers, the formation of distant metastasis was for a long time believed to occur through hematogenous dissemination (Calhoun *et al*., 1994; Leemans *et al*., 1993; Leon *et al*., 2000). However, more recent studies show that distant metastasis without lymph node spread is unusual in oral cell squamous carcinomas, and patients staged N0, N1, N2, and N3 (N0: no spread to lymph nodes; N1: spread to one lymph node; N2: spread to more than one lymph nodes; N3: spread to lymph nodes that are more than 6 cm in size) have a 5‐year survival rate of 63%, 32%, 26%, and 11%, respectively (de Jong *et al*., 2001). Some tumor cells have been shown to migrate into nondraining lymphatic vessels and form tumors, so‐called skip metastases. The incidence of skip metastases was in 1997 reported in 15.8% of 277 tongue cancer cases (Byers *et al*., 1997).

Considering the construction of the lymphatic system, it may not be surprising that this system represents a major route for dissemination of tumor cells. In contrast to blood vessels, lymphatic vessels are built for transport of fluid and cells—away from tissues. Blind‐ended lymphatic capillaries are formed by oak leaf‐shaped lymphatic endothelial cells overlapping each other (Oliver and Detmar, 2002). When the interstitial pressure increases in tissues, the space between the endothelial cells widens, which creates the formation of gaps that allow influx of interstitial fluid (Gerli *et al*., 2000). The creation of this one‐way drainage system, allowing fluid to flow in but not out, was previously interpreted as a defect of endothelial cells lacking intercellular junctions. This theory fell short when gene profiling data demonstrated the expression of endothelial cell–cell adhesion molecules, such as platelet endothelial cell adhesion molecule 1 (PECAM‐1), junction adhesion molecule A (JAM‐A), and occludin (Kriehuber *et al*., 2001; Podgrabinska *et al*., 2002). The final solution of how intercellular junctions are organized in lymphatic capillaries came through high‐resolution imaging studies that demonstrated the existence of button‐like junctions allowing gaps to form and support intravasation of fluid and cells while remaining intact (Baluk *et al*., 2007).

Thus, one reason for why cancer cells may choose lymphatic vessels over blood vessels for intravasation is because lymphatic capillaries are structurally organized to support cellular trafficking away from tissues. Yet, another important question is how cancer cells find their way to lymphatic vessels—do they simply migrate stochastically within the tumor microenvironment until they randomly come in contact with a lymphatic vessel? Or, do mechanisms exist that actively promote targeted migration of cancer cells toward lymphatic vessels?

## 2 Immune cell trafficking through the lymphatic system

Lymphatic vessels are not only serving as a transport system for fluid and cells but represent an important component of the immune system (Kataru *et al*., 2014). The lymphatic system functions as an interface between the innate and adaptive immunity, and actively communicates and senses inflammatory stimuli from the periphery (Shields, 2011). The immune response, which is mounted in draining lymph nodes, plays an important role in coordinating the local response toward the cause of the inflammatory response, and promotes tissue‐specific immunity. An essential part of the process of mounting of an adaptive immune response in lymph nodes is the activation and targeted migration of antigen‐presenting immune cells (APCs) from the periphery and through the lymphatic system. Important cells in this respect are dendritic cells (DC) that possess phagocytic and antigen presentation abilities (Randolph *et al*., 2005). Although DCs are not the only migratory cells, they are at the core of the activation of the adaptive immune system. At steady state, they are also important in promoting peripheral tolerance and thus have a dual role (Steinman and Nussenzweig, 2002). Upon activation, DCs acquire migratory properties characterized by distinct polarization of cellular actin machineries (Vargas *et al*., 2016). The migratory capacity of DCs is influenced by their subtype and by inflammatory cues (Worbs *et al*., 2017). Activated DCs in peripheral tissues do not migrate randomly but rather, in a targeted fashion toward lymphatic vessels, and further toward draining lymph nodes. An important driver of the targeted migration of DCs through the lymphatic system is the chemokine CCL21, which is produced and secreted by lymphatic endothelial cells, and its receptor CCR7, which is expressed at the surface of activated DCs. Through their surface expression of CCR7, activated DCs have the capacity to sense and migrate toward lymphatic endothelial cells expressing CCL21.

To generate an adaptive immune response that includes antibody production, antigens also need to reach the follicular dendritic cells (FDCs) that reside in the B‐cell follicles and are important for B‐cell activation. In this respect, a number of mechanisms are involved including active transport by phagocytes to the lymph nodes (Heesters and Carroll, 2016). However, studies have also shown that small soluble antigens can reach lymph nodes even without antigen‐presenting cells (Sixt *et al*., 2005). Such antigens are processed and transported by subcapsular sinus macrophages (SSM) and B cells to FDCs. The SSM are tissue‐resident macrophages and are positioned so that they can stretch between the lymphatic endothelial cells to capture antigen. Alternatively, antigens may flow into the conduit network, which makes up a tubular system within lymph nodes allowing antigens below 70 kD to enter and be taken up by resident phagocytes and FDCs (Gonzalez *et al*., 2011). After the B cell has picked up its specific antigen from the FDC, it is processed and presented to T cells activated by DCs. This is a key event and the start of the germinal center reaction, which leads to clonal selection and subsequently specific antibody production. Activated effector lymphocytes can exit the lymph nodes and disseminate into blood vessels (Weisel *et al*., 2016). These cells can subsequently traffic to the inflamed site to perform cytotoxicity or, in the case of B cells, remain in secondary lymphoid organs to produce antibodies, or migrate to the bone marrow to become long‐lived plasma cells (Roth *et al*., 2014). Thus, immune cells interact with lymphatic vessels in tissues and lymph nodes. They also secrete factors that may induce remodeling of the lymphatic system through lymphangiogenesis, which is frequently observed in chronic inflammatory and cancer diseases (Kataru *et al*., 2014). Expansion of the lymphatic vasculature in tumors through lymphangiogenesis is linked to enhanced lymph metastasis (Ji, 2012; Zumsteg and Christofori, 2012).

### 2.1 EMT in cancer metastasis

Epithelial‐mesenchymal transition is a developmental process whereby epithelial cells transdifferentiate into mesenchymal cells, migrate to other regions of the embryo, and form new cell types (Nieto *et al*., 2016). Similar to other developmental processes, like angiogenesis and lymphangiogenesis, EMT is silent in normal, healthy tissues. However, reactivation of EMT occurs in pathological conditions including chronic inflammation, fibrosis, wound healing, and cancer (comprehensively reviewed in (Kalluri and Weinberg, 2009; Nieto *et al*., 2016; Thiery *et al*., 2009). Epithelial cells undergoing EMT lose epithelial characteristics, such as components of tight and adherens junctions, and apico‐basal cell polarity, and the cytoskeleton is reorganized to support a more migratory state. Loss of E‐cadherin expression, one of the most frequently used markers for EMT, is associated with a more invasive phenotype in many types of human carcinomas (Frixen *et al*., 1991). The regulation of E‐cadherin has been widely studied, and various transcription factors known for inducing EMT, including Snail1/2, ZEB1/2, and Twist1/2 factors, function as direct or indirect repressors of E‐cadherin (Lamouille *et al*., 2014; Nieto and Cano, 2012). The expression of these EMT factors is regulated by signaling pathways involved in cellular stemness and transformation, including Ras, Notch, and WNT signaling, and is often associated with poor prognosis and tumor recurrence (Elloul *et al*., 2005; Fuxe *et al*., 2010; Martin *et al*., 2005; Moody *et al*., 2005). Loss of tight junction proteins including the coxsackie and adenovirus receptor (CXADR) and occludin, are other hallmarks of EMT, and contributes to loss of the epithelial barrier (De Craene and Berx, 2013; Fuxe *et al*., 2010; Vincent *et al*., 2009). Increased expression of mesenchymal proteins, such as the intermediate filament protein vimentin, contributes to the enhanced migratory properties of EMT cells (Lamouille *et al*., 2014).

In addition to being more migratory, EMT cells display other properties associated with cancer progression and metastasis. As such, EMT cells can avoid senescence. Twist factors can disarm oncogene‐induced senescence by inhibiting the tumor suppressor proteins p16 and p21, alongside inducing EMT (Ansieau *et al*., 2008). Furthermore, early studies provided evidence for the role of EMT in drug resistance (Sommers *et al*., 1992). High levels of vimentin were detected in adriamycin‐resistant cell lines, where some cells in a heterogenic population possessed typical EMT features. This suggested that EMT cells have advantages in growth capabilities compared to non‐EMT cells after drug treatment. Drug resistance is often accompanied with EMT and has been reported in several cancer types like breast and pancreatic cancer (Singh and Settleman, 2010). High levels of E‐cadherin are associated with sensitivity to epidermal growth factor receptor (EGFR) kinase inhibitors, whereas the opposite is true for cells with low levels of E‐cadherin and a mesenchymal phenotype. Cells expressing EMT markers have also been shown to have a higher resistance to drugs such as oxaliplatin and paclitaxel (Kajiyama *et al*., 2007; Yang *et al*., 2006). Recent studies using animal models emphasize the role of EMT as an important mechanism of chemoresistance (Fischer *et al*., 2015; Shibue and Weinberg, 2017; Zheng *et al*., 2015).

### 2.2 Inflammation as an EMT inducer

Recently, much attention has been drawn to the inflammatory milieu within tumors, and infiltrating immune cells have been found to play both anti‐ and protumorigenic roles. Subtypes of tumor‐associated macrophages (TAMs), regulatory T cells, and myeloid‐derived suppressor cells (MDSC) have been identified as promoters of tumor progression and metastasis. These cells produce and secrete cytokines such as transforming growth factor beta (TGF‐β), which may induce EMT, and contribute to an immunosuppressive tumor microenvironment by switching the polarization of immune cells (Fuxe and Karlsson, 2012).

Transforming growth factor beta‐β is frequently overexpressed in cancer and inflammatory tissues (Lamouille *et al*., 2014; Massague, 2008; Moustakas and Heldin, 2016). The TGF‐β paradox refers to its function both as a tumor suppressor and as a promoter. Normally, TGF‐β controls tissue homeostasis by coordinating apoptosis, and inhibits incipient tumor growth (Massague, 2008). However, during tumor progression, TGF‐β switches from a tumor suppressor to a promoter of EMT and resistance to cell death. Breast cancer cells treated with TGF‐β for several weeks stay in EMT and avoid apoptosis (Gal *et al*., 2008). TGF‐β signals through a canonical Smad signaling pathway, which involves phosphorylation and activation of receptor‐Smads 2/3, which then interact with Smad4. The Smad complex enters the cell nucleus, where it regulates the transcription of TGF‐β target genes. However, Smads have low affinity for DNA binding and need to interact with cofactors to achieve target gene specificity (Massague, 2008). The identification of Snail1, a master regulator of EMT, as a cofactor for Smad3/4 opened up a new understanding of how TGF‐β cooperates with oncogenic pathways like Ras‐MAPK, Notch, and WNT/β‐catenin to induce EMT (Fuxe *et al*., 2010; Vincent *et al*., 2009). Interestingly, TGF‐β can also promote endothelial/mesenchymal transition (EndoMT), which also requires Snail1 and Smad transcription factors (Kokudo *et al*., 2008; Mihira *et al*., 2012). EndoMT may contribute to the generation of fibroblasts/myofibroblasts in fibrotic diseases (Piera‐Velazquez *et al*., 2016), but its role in cancer metastasis is not clear.

### 2.3 EMT cells hijack the immune system to disseminate through the lymphatic system

It has not been clear what drives cancer cells to disseminate through the lymphatic system, and whether EMT contributes to this process. However, recent data have shed some new light on this question. Our experiments demonstrated that cancer cells that undergo TGF‐β‐induced EMT acquire an enhanced capacity to disseminate through the lymphatic system (Pang *et al*., 2016). Using a novel three‐dimensional coculture system, in which EMT cells grown on polymeric beads were analyzed for their capacity to migrate toward beads coated with either blood or lymphatic endothelial cells, we found that EMT cells preferentially migrate toward lymphatic compared to blood endothelial cells. These data indicated that lymphatic endothelial cells may produce chemotactic factors that attract EMT cells. Considering the well‐known role of the chemokine receptor CCR7 and its ligand CCL21 in promoting dissemination of DCs through the lymphatic system, we studied whether CCR7/CCL21‐mediated chemotaxis could play a role in targeting EMT cells to lymph nodes. The CCR7 receptor is functioning as an activator of DCs and lymphocytes, and plays an important role for targeted DC migration through the lymphatic system to lymph nodes (Platt and Randolph, 2013). CCR7 has also been shown to mediate lymphatic dissemination of cancer cells (Cunningham *et al*., 2010; Shields *et al*., 2007).

**Figure 1:**
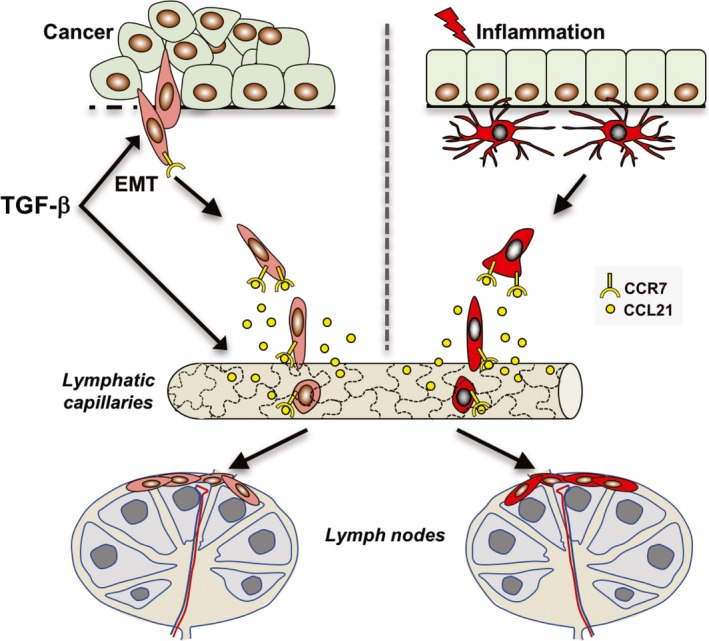
Cancer cells undergoing TGF‐β‐induced EMT acquire properties of immune cells allowing them to disseminate through the lymphatic system similar to DCs during inflammation. Upon activation, DCs acquire cell surface expression of the chemokine receptor CCR7, which allows them to sense and migrate in a targeted fashion toward lymphatic capillaries secreting the CCR7 ligand, CCL21. Endothelial cells of lymphatic capillaries are oak leaf‐shaped and are connected through button‐like junctions, which allows cells to intravasate without junctional disorganization. Subsequently, DCs migrate to lymph nodes where they interact with other cells of the immune system and perform antigen presentation. Recent studies show that similar to activated DCs, cancer cells undergoing TGF‐β‐induced EMT gain the expression of CCR7 and migrate in a targeted fashion through the lymphatic system (Pang *et al*., 2016). TGF‐β may also enhance the migration of EMT cells toward lymphatic capillaries by inducing the expression of CCL21 in lymphatic endothelial cells. It remains to be determined how EMT cells migrate and interact with the immune system within draining lymph nodes.

In our studies, we found that CCR7 was induced in cancer cells undergoing TGF‐β‐induced EMT (Pang *et al*., 2016). Further studies revealed that CCR7 mediated lymph node metastasis of EMT cells. The ligand CCL21 is expressed on lymphatic endothelial cells and serves as an important chemoattractant for migrating DCs. We found that CCL21 produced by lymphatic endothelial cells was important for the capacity of EMT cells to migrate toward lymphatic endothelial cells. Moreover, the data showed that TGF‐β induced the expression of CCL21 in lymphatic, but not in blood endothelial cells. Thus, TGF‐β‐induced EMT in cancer cells appears to activate a mechanism used by the immune system to direct migration of DCs through the lymphatic system (Fig.**** 1). In line with this, others have found that lymphatic endothelial cells may express other chemokines used by tumor cells for lymph node metastasis (Table 1). The CXCR5 receptor and its ligand CXCL13 have been linked to EMT and lymph metastasis in breast cancer (Biswas *et al*., 2014). The chemokine CXCL12 was found to be upregulated in the lymphatic endothelium of draining lymph nodes, alongside migrating tumor cells expressing the CXCR4 receptor (Hirakawa *et al*., 2009), and this system is linked to TGF‐β‐induced EMT (Bertran *et al*., 2009; Taki *et al*., 2008). Another chemokine, CCL1, was shown to promote lymph node entry of melanoma cells expressing the receptor CCR8, whereas blocking of CCR8 function resulted in decreased lymph node metastasis (Das *et al*., 2013). Inhibition of CCR8 additionally prevented tumor cells from lymph node entry, creating an arrest of tumor cells in the afferent lymphatic vessels. Together, these results indicate that TGF‐β‐induced EMT provides cancer cells with the capacity to hijack various chemotactic signals and use them for dissemination through the lymphatic system similar to activated DCs during inflammation.

**Table 1:** Chemokine receptors and ligands linked to TGF‐β‐induced EMT and lymphatic dissemination of cancer cells

Receptors	Ligands	References
CCR7	CCL21	Pang *et al*. (2016)
CCR8	CCL1	Das *et al*. (2013)
CXCR4	CXCL12	Hirakawa *et al*. (2009), Bertran *et al*. (2009), Taki *et al*. (2008)
CXCR5	CXCL13	Biswas *et al*. (2014)

Considering the possibility that cancer cells undergoing EMT may acquire additional properties of immune cells, we performed gene profiling analysis of breast cancer cells that had been allowed to adopt an EMT program after treatment with TGF‐β for 2 weeks. Intriguingly, a cluster of genes, which are normally expressed in myeloid type of immune cells including macrophages and DCs, were induced in the EMT cells (Johansson *et al*., 2015). These genes encode proteins with variable intracellular localization and molecular function, and while some of them previously have been linked to cancer metastasis, others have not. Our conclusion from these studies is that cancer cells undergoing TGF‐β‐induced EMT adopt features of immune cells and that the term ‘mesenchymal’ actually is insufficient to describe the properties of EMT cells. It will be interesting for future studies to further examine to what extent EMT cells make use of immune cell properties to disseminate to lymph nodes and further to distant sites. Intriguingly, the only cells in human bodies that actually share the capacity with prometastatic cancer cells to disseminate through the vascular systems and home to distant organs are immune cells.

## 3 New technical advances to study metastasis

Sites of regional lymph node spread have been termed ‘bridgeheads’, suggesting that they serve as bridges assisting further spread (Sleeman, 2000). However, the mechanisms by which cancer cells migrate both within lymph nodes and further to other sites remain poorly studied. We foresee that the rapidly evolving field of tumor imaging will lead to better insight into this. Macroscopic imaging techniques such as MRI, CT, and PET are mostly applied to clinical practice and allow the visualization of tumors as well as the evaluation of cancer therapy. Microscopic techniques, instead, allow the characterization of molecular mechanisms that underlie metastasis, and cell–cell interactions *in vivo*, which are needed to study the dynamic behavior of metastatic cells (Condeelis and Weissleder, 2010).

Advances in photonics have enabled the development of intravital two‐photon microscopy (IVM), a revolutionary technique that allows the study of cell migration in living organisms at cellular and subcellular resolution (Stein and Gonzalez, 2017). The advantage of this technique, compared to classical imaging methods, resides in the use of low‐energy infrared photons that allow the visualization of cells at a greater specimen depth (typically from 100 to 1000 μm), while minimizing tissue photodamage and photobleaching caused by exposure to laser radiation during the image acquisition process (Warren *et al*., 2003). Moreover, IVM has benefited from the generation of constitutive or inducible fluorescent reporter mouse strains, which have been used for the study of different processes such as the visualization of immune cell interactions (Niesner and Hauser, 2011), the study of the structure and morphology of the lymphatic system (Choi *et al*., 2011), and the oncogenic transformation and tumor regression *in vivo* (Ventura *et al*., 2007). The application of this powerful technology to cancer studies has provided new insights into the spatiotemporal dynamics of tumor progression and the understanding of how the interaction between tumor cells and the stromal compartment influences this process.

**Figure 2:**
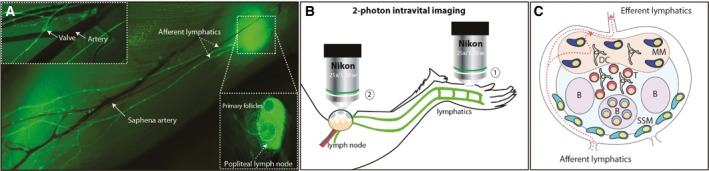
Intravital imaging techniques to study lymphatic dissemination of cancer cells. (A) Photomerge composition of lymphatic and blood circulatory systems of the lower hind limb of a Prox1‐GFP mouse. In the upper box, white arrows indicate a lymphatic valve and the saphena artery. In the main figure, white arrows indicate the afferent vessels, the saphena artery, and the popliteal lymph node (LN). (B) Schematic representation of the surgical intravital two‐photon model in which two different areas of the limb are imaged. On the one hand, the injection site, located in the footpad (1), and on the other hand, the popliteal area, where the arrival of the cancer cells is monitored (2). After administration of anesthesia, the animal is immobilized, the popliteal LN is surgically exposed, and interactions between the cancer and immune cells are recorded using intravital two‐photon microscopy. (C) Schematic representation of the most representative areas and immune cell populations in the popliteal LN. SSM, subcapsular sinus macrophages; MM, medullary macrophages; DC, dendritic cells; B, B‐cell follicle.

However, in order to apply IVM, it is necessary to develop proper microsurgical animal models that combine a minimally invasive surgery, while keeping the organ completely immobilized, which is required for the stable acquisition of micrographs. One of the most commonly used models in IVM is the mouse popliteal lymph node (LN) (Fig.**** 2) developed by Mempel and colleagues more than a decade ago (Mempel *et al*., 2004). The selection of this organ as the model to study cell migration is associated with its prominent lymphatic drainage that originates from the mouse footpad (Fig. 2A), and facilitates the direct observation of migratory cells from the injection site to the LN (Fig. 2B; (1) and (2), respectively) (Stein and Gonzalez, 2017). Additionally, the development of transgenic fluorescent reporter mouse strains, such as the Prox1‐GFP (Choi *et al*., 2011), in which lymphatic endothelial cells are labeled through expression of GFP, has been a valuable tool to study cell migration through the lymphatic system (Fig. 2A).

Despite being one of the key indicators of tumor aggressiveness, the mechanisms of lymphatic dissemination of tumor cells remain elusive. To date, only relatively few studies have focused on the migration of cancerous cells through the lymphatic system using IVM models. Recently, Das *et al*. (2013) monitored, using IVM, the metastasis of melanoma cells in the draining LN. The authors observed that the egress of the tumor cells from the afferent lymphatics to the subcapsular sinus area of the LN is an active mechanism directed by the chemokine CCL1. Secretion of the latter by lymphatic endothelial cells at the junction between the afferent lymphatics and the subcapsular sinus allows the entry of CCR8‐expressing tumor cells into the node and their subsequent metastasis.

Other studies have concentrated in the *in vivo* examination of the encounter of the migratory tumor cell by the immune system. The LN is a highly compartmentalized organ with specific cell groups populating its different areas (Fig. 2C). Among them, subcapsular sinus macrophages (SCS), located next to the afferent lymphatics, act as ‘flypaper’, preventing the dissemination of antigen and pathogens (review in Stein and Gonzalez, 2017). Moalli and colleagues have recently characterized a mechanism by which tumor‐derived antigen (TDA) induces humoral immune responses in tumor‐bearing hosts (Moalli *et al*., 2015). Using IVM, the authors reported that the capture of TDA was associated with SSM and B cells, and they were both involved in the transport of TDA to the follicular dendritic cell network, located inside the follicle. On the other hand, Junankar *et al*. (2015) found that the antitumoral action of bisphosphonates, a drug commonly used for the treatment of osteoporosis, was related to the elimination of tumor‐associated macrophages that facilitate tumor progression (Junankar *et al*., 2015).

Furthermore, other studies focusing on the interactions of effector immune cells with tumor cells in the LN have also been performed using IVM. Deguine and colleagues found that natural killer cells established mainly dynamic contacts with their target cells during tumor regression, while, conversely, cytotoxic T lymphocytes (CTL) formed stable contacts with tumor cells that expressed their cognate antigen (Deguine *et al*., 2010). Similarly, Boisonnas and colleagues imaged the arresting and killing of tumor cells by adoptively transferring CTL that recognized the expression of cognate antigen. Interestingly, the authors found that CTL infiltrate tumors in depth only when the tumor cells express the cognate CTL antigen. Conversely, the infiltration of CTL was restricted to peripheral regions in tumors that do not express the cognate antigen (Boissonnas *et al*., 2007).

These studies have demonstrated the power of IVM as a unique technique to elucidate *in vivo* the mechanisms behind tumor progression. This could also be applied to study the effects on tumors when using checkpoint inhibitors (Sharma and Allison, 2015). Currently, this treatment that targets T cells is changing the way cancer is treated, and there is a drive to enhance the effect of this type of therapy to benefit more patients (Khalil *et al*., 2016). IVM will be important to evaluate how immunotherapy affects infiltration of CTLs and other immune cells in the TME. Besides activation of T‐cell responses, modulation of other immune cells within the stromal compartment including tumor‐associated macrophages (TAM) is an attractive option (Ngambenjawong *et al*., 2017). Also here, IVM could be used to investigate the dynamics of CTL responses when altering the microenvironment. Future IVM studies will provide further insights into the dynamics of lymphatic dissemination and metastasis in different models of cancer, opening the door to the development of novel strategies intended to control the progression of the disease.
